# The serum matrix metalloproteinase-9 level is an independent predictor of recurrence after ablation of persistent atrial fibrillation

**DOI:** 10.6061/clinics/2016(05)02

**Published:** 2016-05

**Authors:** Gang Wu, Shun Wang, Mian Cheng, Bin Peng, Jingjun Liang, He Huang, Xuejun Jiang, Lizhi Zhang, Bo Yang, Yongmei Cha, Hong Jiang, Congxin Huang

**Affiliations:** I Renmin Hospital of Wuhan University, Cardiovascular Research Institute of Wuhan University, Department of Cardiology, Wuhan, Hubei, China; II Mayo Clinic, Department of Medicine, Division of Cardiovascular Diseases, Rochester, MN, USA; III Huazhong University of Science and Technology, Tongji Medical College, Tongji Hospital, Department of Geriatrics, Wuhan, Hubei, China; IV Division of Anatomic Pathology, Department of Laboratory Medicine and Pathology, Mayo Clinic, Rochester, MN, USA

**Keywords:** Matrix Metalloproteinase-9, Atrial Fibrillation, Recurrence, Catheter Ablation

## Abstract

**OBJECTIVES:**

This study investigated whether the serum matrix metalloproteinase-9 level is an independent predictor of recurrence after catheter ablation for persistent atrial fibrillation.

**METHODS:**

Fifty-eight consecutive patients with persistent atrial fibrillation were enrolled and underwent catheter ablation. The serum matrix metalloproteinase-9 level was detected before ablation and its relationship with recurrent arrhythmia was analyzed at the end of the follow-up.

**RESULTS:**

After a mean follow-up of 12.1±7.2 months, 21 (36.2%) patients had a recurrence of their arrhythmia after catheter ablation. At baseline, the matrix metalloproteinase-9 level was higher in the patients with recurrence than in the non-recurrent group (305.77±88.90 *vs* 234.41±93.36 ng/ml, respectively, *p*=0.006). A multivariate analysis showed that the matrix metalloproteinase-9 level was an independent predictor of arrhythmia recurrence, as was a history of atrial fibrillation and the diameter of the left atrium.

**CONCLUSION:**

The serum matrix metalloproteinase-9 level is an independent predictor of recurrent arrhythmia after catheter ablation in patients with persistent atrial fibrillation.

## INTRODUCTION

Catheter ablation is the most promising treatment for atrial fibrillation (AF). The multiple procedure success rate of pulmonary vein isolation (PVI) for paroxysmal AF is approximately 80% after long-term follow-up 1,2. However, the success rates of PVI for persistent AF are low, ranging from 45%-60% 3,4. Recurrences are common after an initial procedure for persistent AF ablation and repeat ablation is often required to maintain freedom from AF 5. Thus, both the outcome and the cost-effectiveness of the ablation procedure are less favorable for persistent than paroxysmal AF. Non-invasive predictors for AF recurrence after catheter ablation are necessary to select the optimal patients for this procedure.

Atrial fibrosis contributes to both electrical and structural atrial remodeling and thrombogenesis in patients with AF. Recent studies have shown that inflammation plays an important role in atrial fibrosis 6,7. Inflammation may also affect the prognosis of patients with AF treated by catheter ablation. In our previous study, a variant of the IL6R gene conferred a risk of AF recurrence after catheter ablation in a Chinese Han population 8. Matrix metalloproteinase-9 (MMP-9), which is a novel fibrotic and inflammatory marker, is correlated with the progression of AF 9. Therefore, we hypothesized that the serum MMP-9 level can also predict recurrence after catheter ablation for persistent AF. In this study, we observed the relationship between the serum MMP-9 level and arrhythmia recurrence after catheter ablation for persistent AF.

## MATERIAL AND METHODS

### Participants

This study recruited 58 consecutive patients (age 55.8±7.9 years, 31 (53.4%) males) with drug-refractory persistent AF who intended to undergo catheter ablation. All patients had documented, recorded, persistent AF. Persistent AF was diagnosed according to the guidelines 10. Patients with structural heart disease; hematological, renal, or hepatic impairment; inflammation; neoplastic disorders; recent (<3 months) myocardial infarction or stroke; acute AF precipitated by thyrotoxicosis; or any acute infection were excluded. Ethical approval was granted by the Institutional Review Board of Renmin Hospital of Wuhan University. All subjects gave written informed consent.

### Blood sampling and echocardiography

The day before catheter ablation, the serum MMP-9 level was detected and trans-esophageal echocardiography (TEE) was performed.

The method used to detect the serum MMP-9 level was described elsewhere 11. Briefly, blood samples were obtained by a peripheral venipuncture and were centrifuged at 3,200 × *g* for 10 minutes at approximately 4°C within an hour of collection. The serum was separated into aliquots and stored at -80°C until personnel blinded to the patients’ clinical information performed the analysis. The serum MMP-9 levels were determined using commercial standardized *in vitro* enzyme-linked immunosorbent assay (ELISA) methods according to the manufacturer’s instructions (RayBiotech Inc, Atlanta, Georgia, USA). The intra- and inter-assay coefficients of variation for the assay were <10% and <12%, respectively.

The left atrial diameter (LAD), left ventricular diameter (LVD) and left ventricular ejection fraction (LVEF) were measured by TEE.

### Catheter ablation for persistent AF

The ablation procedure was performed under local anesthesia. Patients were heparinized to maintain an activated clotting time over 300 s. The atrial anatomy was reconstructed with the CARTO system (Biosense-Webster Inc., Diamond Bar, CA, USA) or NavX mapping system (St. Jude Medical, St. Paul, MN). The ablation procedure comprised the following steps: 1) PVI and 2) linear ablation at the left atrial mitral isthmus and, if the AF was not terminated, the left atrial roof. Additional linear ablation, including the left atrium posterior wall line, right atrium isthmus line and SVC isolation line, was added if the AF was not terminated after steps 1 and 2. The endpoint of the procedure was AF termination. If the AF still did not stop after additional linear ablation, sinus rhythm was restored by electrical cardioversion.

According to the 2012 HRS/EHRA/ECAS expert consensus statement on catheter and surgical ablation of AF, any atrial tachycardia (AT), atrial flutter (AFL) or AF episode lasting longer than 30 seconds should be considered recurrence at three months post-ablation 5.

### Follow-up

All patients were routinely followed up in the outpatient department by cardiologists every month. If patients complained about palpitations, fatigue, or other symptoms related to arrhythmia, Holter monitoring was performed. Patients were also advised to see their doctor anytime they had these symptoms to undergo a 12-lead ECG examination or 24-hour Holter monitoring. In asymptomatic patients, 24-hour Holter monitoring or 7-day cardiac event recording was performed every three months after the procedure. The endpoint for follow-up was documented recurrence of AT/AFL/AF lasting longer than 30 seconds.

### Statistical analysis

All continuous variables are expressed as the means ± SD and categorical variables are expressed as proportions. Between-group comparisons were performed using the two-sample t-test or χ^2^ test as appropriate. Age, sex and variables with *p*<0.1 in the univariate analysis were selected for inclusion in a logistic regression multivariate analysis. The cutoff points for MMP-9 were identified by a receiver operating characteristic (ROC) curve. Statistical significance was established at *p*<0.05. The rates of freedom from AT/AFL/AF were determined and compared using a Kaplan-Meier analysis and log-rank test. The statistical analysis was performed using SPSS software (version 17.0; SPSS Inc., Chicago, IL).

## RESULTS

### Baseline characteristics

After a mean follow-up of 12.1±7.2 months, 21 (36.2%) patients had developed AT/AFL/AF recurrence after catheter ablation. The baseline characteristics of the patients with or without recurrence are shown in Table 1. There were no significant differences in the age, sex, body mass index (BMI), hypertension, LVD, LVEF, or drug use between the two groups. However, patients with recurrent arrhythmia had a longer history of AF, larger LAD and higher serum MMP-9 levels compared with the non-recurrent group.

### Ablation procedure and electrical cardioversion

We retrospectively analyzed the procedure-related data for the non-recurrent and recurrent patients. The rates of AF termination in steps 1, 2 and 3 in the two groups were not significantly different. The only difference between the two groups in terms of the ablation procedure was the use of left atrium roof line ablation. In that stage, the non-recurrent and recurrent groups both had four patients who converted to SR (10.8% * vs* 19.0%, respectively, *p*=0.036) (Table 2).

Six patients in the non-recurrent group and 4 patients in the recurrent group (16.2% *vs* 23.8%, respectively *p*=0.150) who did not covert to SR after total ablation underwent electrical cardioversion (Table 2).

### Logistic multivariate analysis

**Figure 1:**
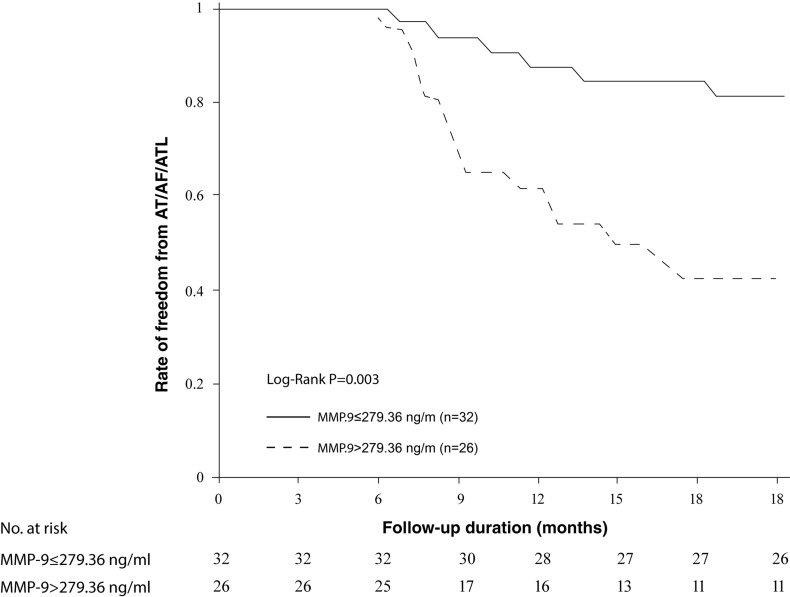
The rates of freedom from AT/AFL/AF, as determined by a Kaplan-Meier analysis. The recurrence rate was higher among patients with a baseline MMP-9 >279.36 ng/ml.

In the logistic multivariate analysis, the MMP-9 levels, AF history and LAD were independent predictors of AF recurrence after catheter ablation for persistent AF (Table 3). Based on the ROC curve, an MMP-9 level >279.36 ng/ml predicted AF recurrence after ablation of persistent AF with a sensitivity of 71.4% and a specificity of 70.3% (area under the ROC curve =0.70). We therefore used 279.36 ng/ml as the cutoff point for the MMP-9 level. The AT/AFL/AF-free survival after catheter ablation, as determined by the Kaplan-Meier curves, showed that there were different AT/AFL/AF survival periods for groups of patients with different MMP-9 levels (Figure 1).

## DISCUSSION

In the present study, we prospectively explored the predictive value of the MMP-9 level for recurrent arrhythmia after catheter ablation. We found that patients with persistent AF who had high baseline MMP-9 levels had an increased rate of recurrence. The MMP-9 level independently predicted AT/AFL/AF recurrence.

The mechanism underlying AF is complex and AF is often caused by multiple factors 12. Atrial myocytes and fibrotic changes of the connective extracellular matrix (ECM) are both involved in the progression of AF. Fibrosis is caused by an imbalance between the degradation and deposition of the cardiac ECM, representing a nonspecific response to cardiomyocyte necrosis or apoptosis. MMPs, which are a multi-gene family of structurally and functionally homogeneous proteolytic enzymes, regulate ECM turnover and may have a determinant role in the atrial structural remodeling involved in the development and perpetuation of AF 13. Previous experimental studies showed that MMP-9 plays a key role in cardiac remodeling and contributes to chamber dilation and excessive collagen accumulation in both aging hearts and hearts post-myocardial infarction 14,15. Recently, MMP-9 was found to have a close relationship with the initiation and perpetuation of AF. Huxley et al. reported that elevated levels of MMP-9 are independently associated with an increased risk of AF 16. Notably, the level of MMP-9 was correlated with the development of AF. In the progression of idiopathic AF, the MMP-9 levels gradually increased from paroxysmal AF through persistent AF to permanent AF 17. Furthermore, previous studies also showed that MMP-9 is associated with atrial remodeling in AF patients.

Nakano et al.^18^ first demonstrated the close relationship between MMP-9 and AF. They showed that increased expression of MMP-9 may contribute to atrial structural remodeling and atrial dilatation during AF. MMP-9 also participates in atrial remodeling after catheter ablation. Additionally, a significant up-regulation of MMP-9 is associated with a greater reduction in the left atrial size 19. In our study, the MMP-9 levels in patients with persistent AF were similar to the previously reported data 16,17. The patients who developed recurrence had a higher serum MMP-9 level, indicating more severe atrial remodeling and advanced AF. These speculations were confirmed by the longer history of AF and larger LAD in this group.

The efficacy of catheter ablation for patients with persistent AF remains unsatisfactory. Despite adopting new techniques, recent studies have reported that up to 40% of patients have relapsed tachycardia after the initial procedure 20,21. Which patient characteristics can be used to evaluate their prognosis has remained unclear. Various candidates for predicting AF recurrence after catheter ablation have been reported, including age, gender, BMI, ECG, the echocardiographic findings, observations made using cardiovascular magnetic resonance (CMR) and some serum or plasma factors 22-25. Some of these studies conflict and the most precise predictors of recurrence after persistent AF ablation remain uncertain. AF progresses with the aggravation of fibrosis and inflammation. Different inflammatory factors cause focal necrosis of the myocardium, modulate ion channel functionality and then initiate the structural and electrical remodeling of the atrium. MMP-9 is one marker of the fibrosis and inflammation that is associated with atrial remodeling in patients with AF. Elevated levels of MMP-9 are related to the occurrence and maintenance of AF in patients with persistent AF 26. In this study, we investigated the factors that influence the outcomes of persistent AF ablation. Because the ablation and electrical cardioversion data in the two groups (high/low MMP-9) were not significantly different, the different outcomes were not due to differences in the ablation procedure. We observed that traditionally reported factors, such as the AF history and LAD, were also significantly associated with the recurrence of AF. In addition, we found that the serum MMP-9 level was an independent predictor of recurrence. In previous studies, the reported changes in the MMP-9 levels in patients with paroxysmal AF were inconsistent and various diseases may cause inflammation and increase the levels of inflammatory factors. Thus, the patients enrolled in our study were all confirmed to have persistent AF without structural heart disease to eliminate this bias. Our data showed that the serum MMP-9 level was effective in predicting recurrence in this AF cohort.

The MMP superfamily comprises many members. In addition to MMP-9, several other members (MMP-2, MMP-3 AND MMP-7) and tissue inhibitors of MMPs (TIMPs), such as TMP1-3, also have a strong association with the incidence of AF 11,27. In the present study, we focused on MMP-9, so the other MMPs/TIMPs are candidates for further study. Whether any of these MMPs/TIMPs (or their combinations) are also indicators of the risk of AF recurrence after catheter ablation remains of interest.

Our findings may be helpful to select which patients with persistent AF should undergo catheter ablation. Because the MMP-9 level is correlated with atrial fibrosis and predicts AF relapses, it may also represent a therapeutic target. Inhibition of the MMPs and regulation of the extracellular collagen matrix might be useful therapeutically in patients with AF. Gene deletion or pharmacological inhibition of MMP activity attenuates atrial remodeling and decreases the vulnerability to AF 28-30. In the TIPTOP trial, the MMP tissue inhibitor doxycycline was used short-term in patients with acute myocardial infarction and left ventricular dysfunction. The trial results showed that doxycycline therapy inversely correlated with the six-month infarct size and severity and left ventricular dilation 31. No clinical trials regarding the use of MMP tissue inhibitors to treat AF have been reported. A prospective randomized trial to determine the value of the MMP-9 level in predicting AF recurrence and to assess the effects of an MMP tissue inhibitor on persistent AF may be warranted.

The patients enrolled in this study were a special AF cohort. Atrial fibrosis is correlated with the development of AF. Post-mortem biopsies, electroanatomical mapping studies and delayed-enhancement CMR images have demonstrated that there is more extensive fibrosis in patients with persistent AF than paroxysmal AF. In patients with permanent AF, the fibrosis is more extensive than in those with persistent AF 32-37. Permanent AF usually co-exists with structural heart disease, which may aggravate fibrosis and inflammation. In recent guidelines, catheter ablation is not recommended for patients with permanent AF. The present study only evaluated patients with persistent AF without underlying structural disease, which might limit its generalizability.

The serum MMP-9 level was higher in patients with recurrence, and it was identified as an independent predictor of arrhythmia relapse after catheter ablation in patients with persistent AF.

## AUTHOR CONTRIBUTIONS

Wu G designed the study, participated in data acquisition, performed statistical analyses and drafted the manuscript. Wang S, Cheng M, Peng B and Liang J performed the sample collection and serum MMP-9 detection. Zhang L, Cha Y, Jiang H and Huang C contributed to the conception and design of the work and revised the final draft. Huang H and Yang B performed the ablation procedures. Jiang X performed the data analysis. All authors have read and approved the final manuscript for publication.

## Figures and Tables

**Table 1:** Baseline characteristics of the patients with and without recurrence.

	Non-recurrent (N=37)	Recurrent (N=21)	*p* value
Age (years)	54.4±6.9	58.3±7.6	0.057
Gender, male	21 (56.8)	11 (52.3)	0.819
Body mass index (kg/m^2^)	26.3±5.2	27.8±6.4	0.336
AF history (months)	44.6±16.5	54.6±17.9	0.036
Hypertension	5 (13.5)	3 (14.2)	0.888
LAD (mm)	38.6±7.9	45.1±9.4	0.007
LVDD (mm)	51.6±8.3	53.1±7.1	0.490
LVEF (%)	57.1±8.3	57.9±9.6	0.740
ACEI/ARB	7 (18.9)	3 (14.2)	0.358
Amiodarone (n, %)	19 (51.4)	12 (57.1)	0.740
BB (n, %)	34 (91.9)	19 (90.5)	0.995
MMP-9 (ng/ml)	234.41±93.36	305.77±88.90	0.006

Values are given as the No. (%) or means±SD.

**Table 2:** Procedure-related data for the patients with and without recurrence.

	Non-recurrent (N=37)	Recurrent (N=21)	*p* value
Step 1: PVI	7 (18.9)	3 (14.3)	0.329
Step 2: linear ablation of the left atrium	16 (43.2)	12 (57.1)	0.295
left isthmus line	12 (32.4)	8 (38.1)	0.549
left roof line	4 (10.8)	4 (19.0)	0.036
Steps 1 + 2	23 (62.2)	15 (71.4)	0.606
Step 3: additional linear ablation	14 (37.8)	6 (28.5)	0.329
All three steps	31 (83.8)	28 (76.2)	0.082
Electrical cardioversion	6 (16.2)	4 (23.8)	0.150

Values are given as the No. (%). Additional lines included the left atrium posterior wall line, the right atrium isthmus line and the SVC isolation line.

**Table 3:** The results of the multivariate analysis and the 95% confidence intervals for recurrent atrial fibrillation.

	*p* value	Odds ratio	95% confidence interval
Age (years)	0.394	0.981	0.863-1.102
AF history (months)	0.038	1.243	1.082-1.427
LAD (mm)	0.017	1.225	1.024-1.443
MMP-9 (ng/ml)	0.029	1.136	1.018-1.273

## References

[1] Pappone C, Vicedomini G, Augello G, Manguso F, Saviano M, Baldi M (2011). Radiofrequency catheter ablation and antiarrhythmic drug therapy: a prospective, randomized, 4-year follow-up trial: the APAF study. Circ Arrhythm Electrophysiol.

[2] Elayi CS, Verma A, Di Biase L, Ching CK, Patel D, Barrett C (2008). Ablation for longstanding permanent atrial fibrillation: results from a randomized study comparing three different strategies. Heart Rhythm.

[3] Sanders P, Hocini M, Jais P, Sacher F, Hsu LF, Takahashi Y (2007). Complete isolation of the pulmonary veins and posterior left atrium in chronic atrial fibrillation. Long-term clinical outcome. Eur Heart J.

[4] Tilz RR, Rillig A, Thum AM, Arya A, Wohlmuth P, Metzner A (2012). Catheter ablation of long-standing persistent atrial fibrillation: 5-year outcomes of the Hamburg Sequential Ablation Strategy. J Am Coll Cardiol.

[5] Calkins H, Kuck KH, Cappato R, Brugada J, Camm AJ, Chen SA (2012). 2012 HRS/EHRA/ECAS Expert Consensus Statement on Catheter and Surgical Ablation of Atrial Fibrillation: recommendations for patient selection, procedural techniques, patient management and follow-up, definitions, endpoints, and research trial design. Europace.

[6] Hu YF, Chen YJ, Lin YJ, Chen SA (2015). Inflammation and the pathogenesis of atrial fibrillation. Nat Rev Cardiol.

[7] Van Wagoner DR (2008). Oxidative stress and inflammation in atrial fibrillation: role in pathogenesis and potential as a therapeutic target. J Cardiovasc Pharmacol.

[8] Wu G, Cheng M, Huang H, Yang B, Jiang H, Huang C (2014). A variant of IL6R is associated with the recurrence of atrial fibrillation after catheter ablation in a Chinese Han population. PLoS One.

[9] Sonmez O, Ertem FU, Vatankulu MA, Erdogan E, Tasal A, Kucukbuzcu S (2014). Novel fibro-inflammation markers in assessing left atrial remodeling in non-valvular atrial fibrillation. Med Sci Monit.

[10] Wann LS, Curtis AB, Ellenbogen KA, Estes NA, Ezekowitz MD, Jackman WM (2013). Management of patients with atrial fibrillation (compilation of 2006 ACCF/AHA/ESC and 2011 ACCF/AHA/HRS recommendations): a report of the American College of Cardiology/American Heart Association Task Force on practice guidelines. Circulation.

[11] Kalogeropoulos AS, Tsiodras S, Rigopoulos AG, Sakadakis EA, Triantafyllis A, Kremastinos DT (2011). Novel association patterns of cardiac remodeling markers in patients with essential hypertension and atrial fibrillation. BMC Cardiovasc Disord.

[12] Qiu XB, Xu YJ, Li RG, Xu L, Liu X, Fang WY (2014). PITX2C loss-of-function mutations responsible for idiopathic atrial fibrillation. Clinics.

[13] Gramley F, Lorenzen J, Plisiene J, Rakauskas M, Benetis R, Schmid M (2007). Decreased plasminogen activator inhibitor and tissue metalloproteinase inhibitor expression may promote increased metalloproteinase activity with increasing duration of human atrial fibrillation. J Cardiovasc Electrophysiol.

[14] Yabluchanskiy A, Ma Y, Chiao YA, Lopez EF, Voorhees AP, Toba H (2014). Cardiac aging is initiated by matrix metalloproteinase-9-mediated endothelial dysfunction. Am J Physiol Heart Circ Physiol.

[15] Khalili H, Talasaz AH, Salarifar M (2012). Serum vitamin D concentration status and its correlation with early biomarkers of remodeling following acute myocardial infarction. Clin Res Cardiol.

[16] Huxley RR, Lopez FL, MacLehose RF, Eckfeldt JH, Couper D, Leiendecker-Foster C (2013). Novel association between plasma matrix metalloproteinase-9 and risk of incident atrial fibrillation in a case-cohort study: the Atherosclerosis Risk in Communities study. PLoS One.

[17] Li M, Yang G, Xie B, Babu K, Huang C (2014). Changes in matrix metalloproteinase-9 levels during progression of atrial fibrillation. J Int Med Res.

[18] Nakano Y, Niida S, Dote K, Takenaka S, Hirao H, Miura F (2004). Matrix metalloproteinase-9 contributes to human atrial remodeling during atrial fibrillation. J Am Coll Cardiol.

[19] Richter B, Gwechenberger M, Socas A, Zorn G, Albinni S, Marx M (2011). Time course of markers of tissue repair after ablation of atrial fibrillation and their relation to left atrial structural changes and clinical ablation outcome. Int J Cardiol.

[20] Mont L, Bisbal F, Hernandez-Madrid A, Perez-Castellano N, Vinolas X, Arenal A (2014). Catheter ablation vs. antiarrhythmic drug treatment of persistent atrial fibrillation: a multicentre, randomized, controlled trial (SARA study). Eur Heart J.

[21] Wynn GJ, Das M, Bonnett LJ, Panikker S, Wong T, Gupta D (2014). Efficacy of catheter ablation for persistent atrial fibrillation: a systematic review and meta-analysis of evidence from randomized and nonrandomized controlled trials. Circ Arrhythm Electrophysiol.

[22] Combes S, Jacob S, Combes N, Karam N, Chaumeil A, Guy-Moyat B (2013). Predicting favourable outcomes in the setting of radiofrequency catheter ablation of long-standing persistent atrial fibrillation: a pilot study assessing the value of left atrial appendage peak flow velocity. Arch Cardiovasc Dis.

[23] Scaglione M, Gallo C, Battaglia A, Sardi D, Gaido L, Anselmino M (2014). Long-term progression from paroxysmal to permanent atrial fibrillation following transcatheter ablation in a large single-center experience. Heart Rhythm.

[24] Yuen HC, Roh SY, Lee DI, Ahn J, Kim DH, Shim J (2015). Atrial fibrillation cycle length as a predictor for the extent of substrate ablation. Europace.

[25] Sramko M, Peichl P, Wichterle D, Tintera J, Weichet J, Maxian R (2015). Clinical value of assessment of left atrial late gadolinium enhancement in patients undergoing ablation of atrial fibrillation. Int J Cardiol.

[26] Lewkowicz J, Knapp M, Tankiewicz-Kwedlo A, Sawicki R, Kaminska M, Waszkiewicz E (2015). MMP-9 in atrial remodeling in patients with atrial fibrillation. Ann Cardiol Angeiol (Paris).

[27] Mukherjee R, Akar JG, Wharton JM, Adams DK, McClure CD, Stroud RE (2013). Plasma profiles of matrix metalloproteinases and tissue inhibitors of the metalloproteinases predict recurrence of atrial fibrillation following cardioversion. J Cardiovasc Transl Res.

[28] Moe GW, Laurent G, Doumanovskaia L, Konig A, Hu X, Dorian P (2008). Matrix metalloproteinase inhibition attenuates atrial remodeling and vulnerability to atrial fibrillation in a canine model of heart failure. J Card Fail.

[29] Chen CL, Huang SK, Lin JL, Lai LP, Lai SC, Liu CW (2008). Upregulation of matrix metalloproteinase-9 and tissue inhibitors of metalloproteinases in rapid atrial pacing-induced atrial fibrillation. J Mol Cell Cardiol.

[30] Lombardi F, Belletti S, Battezzati PM, Pacciolla R, Biondi ML (2011). MMP-1 and MMP-3 polymorphism and arrhythmia recurrence after electrical cardioversion in patients with persistent atrial fibrillation. J Cardiovasc Med (Hagerstown).

[31] Cerisano G, Buonamici P, Gori AM, Valenti R, Sciagra R, Giusti B (2015). Matrix metalloproteinases and their tissue inhibitor after reperfused ST-elevation myocardial infarction treated with doxycycline. Insights from the TIPTOP trial. Int J Cardiol.

[32] Hirsh BJ, Copeland-Halperin RS, Halperin JL (2015). Fibrotic atrial cardiomyopathy, atrial fibrillation, and thromboembolism: mechanistic links and clinical inferences. J Am Coll Cardiol.

[33] Boldt A, Wetzel U, Lauschke J, Weigl J, Gummert J, Hindricks G (2004). Fibrosis in left atrial tissue of patients with atrial fibrillation with and without underlying mitral valve disease. Heart.

[34] Kottkamp H (2013). Human atrial fibrillation substrate: towards a specific fibrotic atrial cardiomyopathy. Eur Heart J.

[35] Platonov PG, Mitrofanova LB, Orshanskaya V, Ho SY (2011). Structural abnormalities in atrial walls are associated with presence and persistency of atrial fibrillation but not with age. J Am Coll Cardiol.

[36] Teh AW, Kistler PM, Lee G, Medi C, Heck PM, Spence SJ (2012). Electroanatomic remodeling of the left atrium in paroxysmal and persistent atrial fibrillation patients without structural heart disease. J Cardiovasc Electrophysiol.

[37] Oakes RS, Badger TJ, Kholmovski EG, Akoum N, Burgon NS, Fish EN (2009). Detection and quantification of left atrial structural remodeling with delayed-enhancement magnetic resonance imaging in patients with atrial fibrillation. Circulation.

